# Change in PM_2.5_ exposure and mortality among Medicare recipients: Combining a semi-randomized approach and inverse probability weights in a low exposure population

**DOI:** 10.1097/EE9.0000000000000054

**Published:** 2019-06-10

**Authors:** Yara Abu Awad, Qian Di, Yan Wang, Christine Choirat, Brent A. Coull, Antonella Zanobetti, Joel Schwartz

**Affiliations:** a Department of Environmental Health, Harvard T.H. Chan School of Public Health, Boston, Massachusetts; b Department of Psychology, Concordia University, Montreal, Canada; c Research Center for Public Health, Tsinghua University, Beijing, China; d Department of Biostatistics, Harvard T.H. Chan School of Public Health, Boston, Massachusett; e Swiss Data Science Center, Lausanne, Canton of Vaud, Switzerland; f Department of Epidemiology, Harvard T.H. Chan School of Public Health, Boston, Massachusetts.

## Abstract

The association between PM_2.5_ and mortality is well established; however, confounding by unmeasured factors is always an issue. In addition, prior studies do not tell us what the effect of a sudden change in exposure on mortality is. We consider the sub-population of Medicare enrollees who moved residence from one ZIP Code to another from 2000 to 2012. Because the choice of new ZIP Code is unlikely to be related with any confounders, restricting to the population of movers allows us to have a study design that incorporates randomization of exposure. Over 10 million Medicare participants moved. We calculated change in exposure by subtracting the annual exposure at original ZIP Code from exposure at the new ZIP Code using a validated model. We used Cox proportional hazards models stratified on original ZIP Code with inverse probability weights (IPW) to control for individual and ecological confounders at the new ZIP Code. The distribution of covariates appeared to be randomized by change in exposure at the new locations as standardized differences were mostly near zero. Randomization of measured covariates suggests unmeasured covariates may be randomized also. Using IPW, per 10 µg/m^3^ increase in PM_2.5_, the hazard ratio was 1.21 (95% confidence interval [CI] = 1.20, 1.22] among whites and 1.12 (95% CI = 1.08, 1.15) among blacks. Hazard ratios increased for whites and decreased for blacks when restricting to exposure levels below the current standard of 12 µg/m^3^. This study provides evidence of likely causal effects at concentrations below current limits of PM_2.5_.

**What this study adds**

To our knowledge, this is the first study looking at the effect of change in PM_2.5_ exposure due to moving on risk of mortality. Our findings, which used Medicare data, show that per 10 µg/m^3^ increase in exposure, the hazard rate was 1.21 among white movers and 1.12 among black movers. Associations persisted below current limits of PM_2.5_. Key strengths of our analysis are that we studied the entire national Medicare population who moved, including people living in smaller towns and rural areas that have been underrepresented in most cohort studies and the use of causal modeling techniques.

## Introduction

Multiple studies have found an association between ambient PM_2.5_ and mortality, including cardiovascular and lung-cancer mortality following both acute^1,2^ and chronic^3–7^ exposure. These associations have also persisted below the current PM_2.5_ standard of 12 µg/m^3^.^8,9^ Furthermore, there is evidence that race is a modifier of this relationship.^8,10^ One criticism of previous studies on this topic; however, is that they are observational, and although they adjust for confounding, inferring causality is more difficult.

Causal modeling, by contrast, seeks to make data from an observational study closely approximate that from a randomized controlled trial whose causal interpretation is widely accepted. In epidemiology, causal methods for eliminating confounding bias, such as standardization and inverse probability weighting, make the assumption of exchangeability; which is that conditioning on all confounders renders the exposed exchangeable with the unexposed.^11^ Another approach to control for confounding bias via methods such as instrumental variables, regression discontinuity analyses, difference in differences, and natural experiments relies on the exclusion restriction assumption.^12^ This assumption assumes that an event/variable is causing variation in exposure that is not associated with any measured or unmeasured confounders.

There are several examples of natural experiments (i.e., events that lead to variation in exposure) that have been used to study the effects of air pollution. One example is a study by Pope et al^13^ which took advantage of the sudden reductions in PM_10_ in Utah valley when a steel mill closed in order to look at the changes in hospital admissions. The study found that, relative to the time during closure, during mill operation these admissions doubled among children and increased by 47% among adults. More recently, a article by Currie and Walker^14^ looked at the effect of reductions of traffic pollution due to the staggered introduction of electronic toll collection (which was effectively random), and found reductions in premature births and low infant birth weight among women living within 2 km of each toll booth.

These events are rare and it may be difficult to capture the effects without prior knowledge of their occurrence. An easier way to capture the effects of a sudden change in exposure was demonstrated by Avol et al.^15^ In this study, the authors looked at the effect of change in PM_10_ exposure on lung function among the child participants of a longitudinal prospective cohort who moved during the study. They found that those who moved to a location with higher exposure showed decreased growth in lung function and vice versa.

This is an especially interesting approach because restricting an analysis to people who move allows us to study the effect of sudden change in exposure (given that the exposure at the new residence is different). This is rarely observed among people who do not move as year-to-year exposures at the same location are highly correlated.

Additionally, if we assume that, conditional on the decision to move, the choice of a new residence is independent of air pollution, then post-move exposure is uncorrelated with measured and unmeasured covariates that cause mortality, a causal effect can be estimated. We believe this assumption is reasonable for most confounders because movers are generally unaware of the PM_2.5_ concentrations at the locations they consider moving to.

In this article, we applied 3 approaches to addressing causal modeling. First, we looked at the change in exposure between old and new ZIP Code as our exposure under the assumption that the change is independent of measured and unmeasured confounders. Second, we stratified our analysis on old ZIP Code, so that all confounders. Measured or unmeasured, that exist on the neighborhood level are controlled, including past exposure. And finally, we used a propensity score model for individual level and ZIP Code level covariates at the new ZIP Code to create inverse probability weights to control for those potential confounders after the move.

We explored the assumption that moving leads to exposure randomization by comparing the distribution of confounders by change in exposure among people who moved. We carried out this analysis separately among persons with white and black race (herein referred to as whites and blacks) due to the aforementioned effect modification by race and because residential segregation in the United States makes it difficult to balance some neighborhood covariates when both races are combined. To examine effects at lower concentrations, we performed an additional analysis restricted to persons with exposure at their new ZIP Code of 12 µg/m^3^ or lower.

## Methods

### Study population

We extracted records from a database of all Medicare enrollees (ages 65 and over) from 2000 to 2012 residing in the contiguous United States. We identified 12,131,927 movers (persons whose ZIP Code had changed at least once at any time during the study period), and after excluding 36,409 persons missing race and 14 with unknown gender, we were left with 12,095,504 people.

### Covariates

Age, race, sex, and ZIP Code of residence were available for enrollees, in addition to whether the participant was eligible for Medicaid, a supplemental coverage for low-income individuals which is an indicator of socioeconomic status. We used ICD-9 discharge diagnoses to identify whether or not an enrollee had been hospitalized with the following conditions as a primary or secondary cause before they moved: Alzheimer’s disease, acute myocardial infarction, diabetes Mellitus, heart failure, Parkinson’s disease, pneumonia, other respiratory diseases, ischemic stroke, unstable angina, vascular dementia, chronic obstructive pulmonary disease and lung cancer.

Additionally, we obtained ZIP Code level variables from the American Community Survey and the 2000 and 2010 U.S. Census. These include median household income, population density, percentage black, percentage of owner-occupied housing units, median value of owner-occupied housing, percentage above age 65 living below the poverty level, and percentage above age of 65 with less than high school education. We linearly interpolated any missing values that occurred between 2 years.

### Exposure assessment

Mean annual exposure to PM_2.5_ for each enrollee at his/her residential ZIP Code for each year between 2000 and 2012 was estimated using a neural network based hybrid prediction model which is described in detail elsewhere.^16^ In brief, the model used data from multiple sources including predictions of the chemical transport model GEOS-Chem, meteorological data and land-use terms in addition to satellite-based aerosol optical depth, surface reflectance, and absorbing aerosol index data. These variables were used to train a neural network to United States Environmental Protection Agency (EPA) Air Quality System monitoring data from the continental United States in order to generate daily PM_2.5_ predictions on a 1 × 1 km grid. The model showed good performance with ten-fold cross validation yielding an R^2^ of 0.84.

Daily predictions were generated and then averaged over the calendar year for the 4 grids closest to the centroid of the ZIP Code of residence. A mover’s ZIP Code of residence for each calendar year was provided in the Medicare dataset but the exact date of move within the year was not available. It was therefore unclear what proportion of the calendar year was spent at the new ZIP Code versus the ZIP Code of original residence. In order to avoid exposure misclassification, we excluded the first year at the new ZIP Code and began our analysis in the second calendar year at the new ZIP Code. This reduced the sample to 10,679,150 individuals. Of these, about 9 million were white and about 900,000 were black.

Change in exposure was calculated by subtracting the mean annual exposure in the last year at the old ZIP Code from the mean annual exposure in the first full calendar year at the new ZIP Code. We opted to use the difference in mean annual exposure because we wanted to focus on the effect of change in exposure on change in mortality risk.

### Statistical methods

We first checked if moving randomizes exposure by examining whether movers who experienced change in exposure greater than the median were exchangeable with those who saw a change exposure less than the median. We did this by calculating standardized differences in both individual and ZIP Code level covariates (measured at the new ZIP Code) among these 2 exposure groups. The standardized differences were estimated using the equations proposed by Austin.^17^


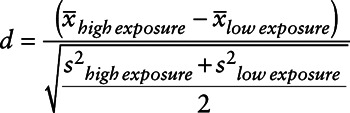
(1)


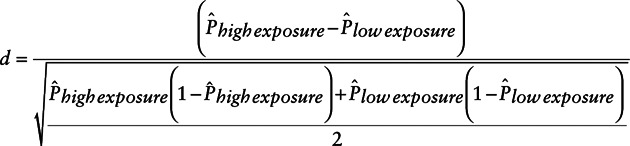
(2)

The standardized differences in continuous confounders (equation 1) and means of dichotomous confounders (equation 2) were calculated among blacks and whites separately. This was repeated for change in exposure above and below the 90th percentile.

To deal with potential confounding by covariates at the new ZIP Code, we fit a propensity score model to estimate inverse probability weights (IPW). Inverse Probability Weighing is a method that allows for the estimation of causal effects from observational data by applying weights to the population so that the exposure is no longer associated with confounders. These weights are the inverse conditional probabilities of exposure given the covariates. Since we have a continuous exposure, we used a generalized propensity score model to calculate these conditional probabilities.^18^ We opted to use stabilized weights as the unstabilized weights for continuous exposures can have infinite variance and are therefore unusable.^11^ Weights were stabilized using the marginal probability of exposure as a numerator as follows:


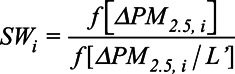


where 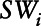 is the stabilized inverse probability weight for the *ith* mover, 
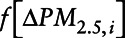
 is the marginal density function of change in exposure evaluated at the observed change in exposure for the *ith* mover, 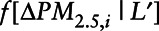 is the conditional density function of change in exposure where the vector of covariates is denoted by the vector *L*′, evaluated at observed covariate values for mover *I*.

Specifically, the numerator of the weights was estimated with an intercept only model:


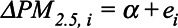


In order to estimate the denominator, we fit the following generalized propensity score model (among whites and blacks separately) which contains both individual and ZIP Code level covariates:


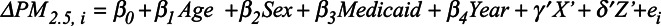


where 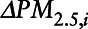 is the *ith* mover’s change in PM_2. 5_ exposure (mean annual PM_2.5_ in second calendar year at new ZIP Code – mean annual PM_2.5_ in year before move); *Age* is the participant’s age in the first year after move; *Sex* is the participant’s sex; 
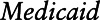
 is an indicator variable for eligibility for Medicaid coverage, and a proxy for lower socioeconomic position (SEP); 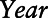 is an indicator variable for calendar year *k*; 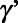 is a vector of coefficients; 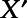 is a vector of indicator variables for hospitalization for any of the 13 conditions mentioned above if they occurred before the move; 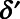 is a vector of coefficients; 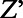 is a vector of the following socioeconomic variables at the *new* ZIP Code: % black residents, % Hispanic residents, median household income, median house value, % occupants who own home, population density, % aged over 65 below poverty level and % aged over 65 with no high school education.

As illustrated by Hirano and Imbens^19^ the residuals of this model can be used to estimate the conditional probability of exposure. Conceptually, because the residuals represent the variance in exposure that cannot be explained by covariates, the distribution of the residuals of this model represents the probability density of receiving the exposure each subject got, given the values of the covariates.

We included hospital admissions before move in our model, as we believe that poor health is a potential confounder of the relationship between change in PM_2.5_ exposure and mortality.

To ensure that the model was correctly specified, we checked the distribution of the continuous variables and log-transformed those that appeared log normal (median household income, median house value, and population density). We also used splines for the following continuous variables: age, % aged over 65 below poverty level, % aged over 65 with no high school education and % black residents. Finally, we added interaction terms for dual eligibility, calendar year, and gender.

To avoid the assumption that the residuals are normally distributed, we used a kernel density estimator on the residuals of both models in order to model the density functions of the marginal and conditional probabilities of exposure, and then calculated the marginal and conditional probability of exposure for each mover and divided one by the other. The optimal smoothing bandwidth was selected based on obtaining “well-behaved weights” (mean = 1, small range).

In order to avoid the use of over-inflated weights,^20^ we assigned the 99th percentile weight to any weights at the 99th percentile or greater and the 1st percentile weights to any weights at the 1st percentile or lower.

We then estimated the effect of change in exposure on risk of all-cause mortality using a Cox proportional hazards model stratified by ZIP Code before move with inverse probability weighting to account for confounding after move and follow-up time as a time axis. Specifically we fit:


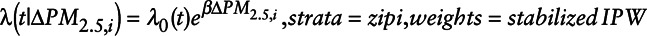


where *t* is the follow-up time which began 1 year after move until either death or censoring; *zip*_*i*_ is the ZIP Code before move.

Stratification by ZIP Code of origin assures that the comparison between exposure change and mortality is only done among people originating from the same neighborhood who moved to different locations. This eliminates confounding by any unmeasured neighborhood level confounder, including past PM_2.5_ exposure at the ZIP Code of origin. A robust variance estimator was applied in order to account for the use of weights.

Finally, we repeated all analyses among black and white movers with exposures of 12 µg/m^3^ or less.

## Results

Our sample of movers had a mean age of 77.1 in the year before their move. About 60% were female and 85% were white (Table 1).

**Table 1:**
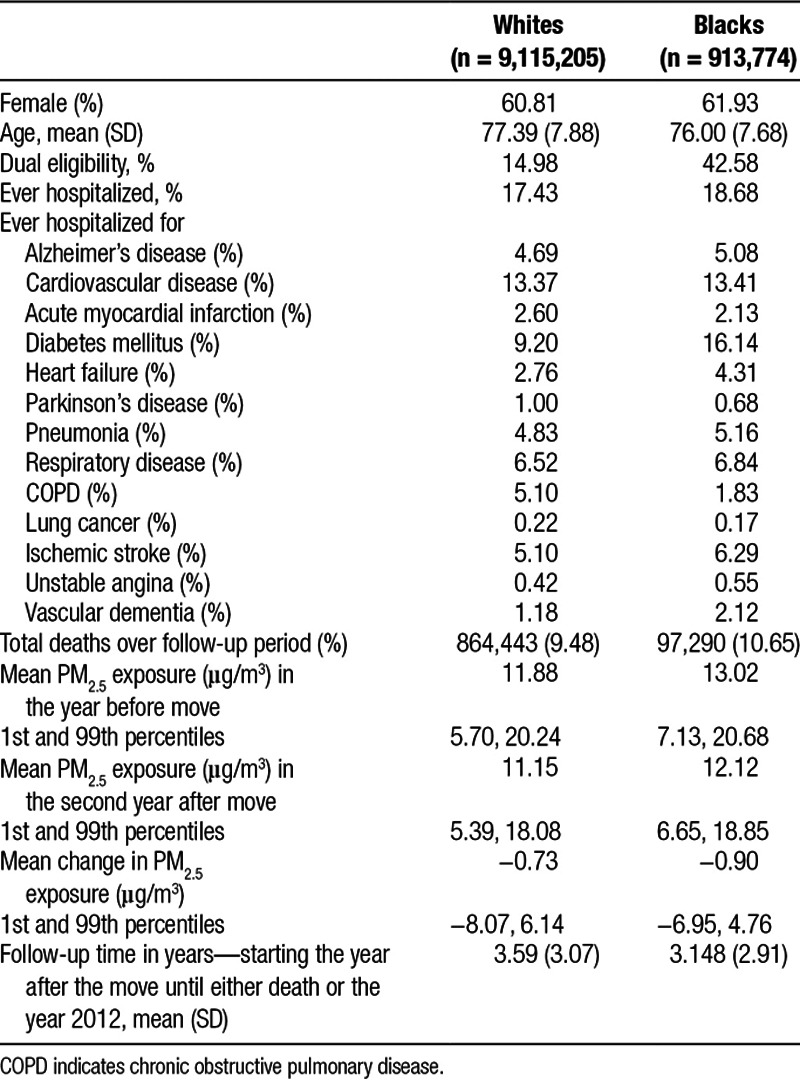
Characteristics of black and white movers in the Medicare cohort

**Figure 1.**
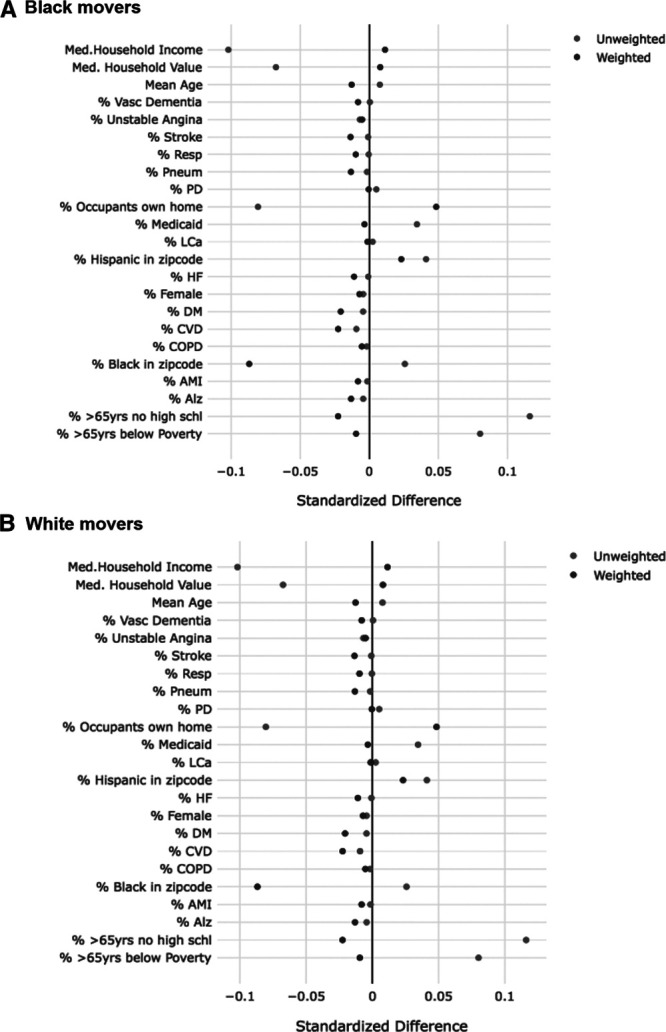
Standardized differences in both individual and ZIP Code level covariates at the new ZIP Code comparing movers with change in exposure above the median* to those with change in exposure below the median in weighted and unweighted populations. A, Black movers. B, White movers. *Median change in exposure is −0.9 µg/m^3^ among blacks and −0.7 µg/m^3^ among whites.

**Figure 2.**
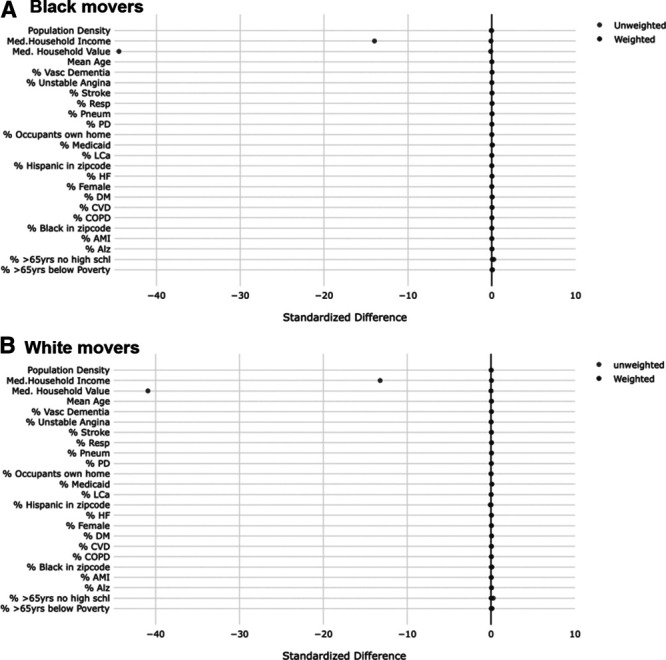
Standardized differences in both individual and ZIP Code level covariates at the new ZIP Code comparing movers with change in exposure above the 90th percentile* to those with change in exposure below the 90th percentile in weighted and unweighted populations. A, Black movers. B, White movers. *90th percentile change in exposure is 1.36 µg/m^3^ among blacks and 2.07 µg/m^3^ among whites.

Comparing the standardized differences in individual and ZIP code level (at the new ZIP code) covariates of movers whose PM_2.5_ change in exposure was above versus below the median (median = −0.90 µg/m^3^ among blacks and −0.69 µg/m^3^ among whites) before and after the use of IPW (Figure 1), it appears that overall, moving reduces these differences to 0.1 or less (on the absolute scale), indicating good balance (i.e., moving appears to have randomized exposure with respect to the covariates considered). After applying the IPW, these standardized differences became even smaller. This supports our theory that moving seems to randomize a person’s exposure. When comparing those with change in exposure at the 90th percentile or greater to those below, it appears that most covariates are balanced with the exception of median household income and median house value (Figure 2). The use of weights for these variables restored balance, and all differences were 0.12 or lower on the absolute scale in the weighted populations.

Most movers (73.4%) moved within their own state and median follow-up time after moving was 4 years. Approximately 61% of the cohort was exposed to PM_2.5_ concentrations below 12 µg/m^3^ at the new location in the second calendar year after move. On average, exposure decreased by 0.91 µg/m^3^ among blacks, but the change in exposure ranged from a decrease of 21.33 µg/m^3^ to an increase of 24.63 µg/m^3^. This was similar among whites, where the mean decrease in exposure was 0.73 µg/m^3^ and the change ranged from −25.10 to 26.83 µg/m^3^.

The IP weights we constructed for our analysis had a mean of 1.01 with a SD of 0.34 among whites and a mean of 0.99 and SD of 0.47 in the black subset. Truncation did not significantly change these means or SDs.

Cox model estimates are presented in Table 2. We found that white movers appear more responsive to a change in exposure (harzard ratio [HR] = 1.21; 95% CI = 1.20, 1.22 per 10 µg/m^3^ increment) compared to black movers (HR = 1.12; 95% CI = 1.08, 1.15). The associations persist among movers whose new ZIP Code had an ambient PM_2.5_ concentration of 12 µg/m^3^ or lower, with a higher HR for whites, and a slightly lower HR for blacks.

**Table 2:**
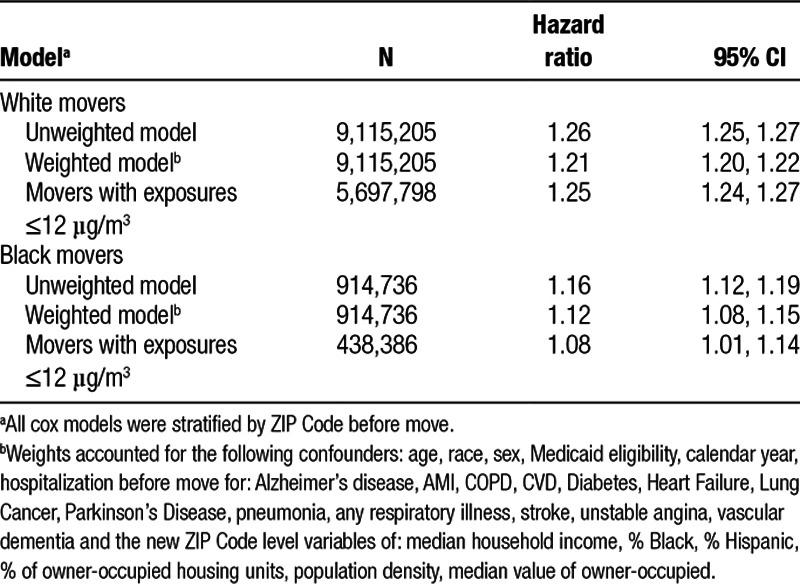
Hazard ratios of all-cause mortality associated with a 10 µg/m^3^ increase in 1-year mean PM_2.5_ in the second year after move

## Discussion

To our knowledge, this is the first study taking advantage of the natural experiment created by moving in addition to confounding adjustment via IPW in such a large population in order to estimate the effect of change in PM_2.5_ on mortality using causal modeling. The balance plot shows that moving balanced all measured covariates. While this balancing can only be examined for measured covariates, the pattern provides support for the notion that moving likely balanced some unmeasured covariates as well, strengthening the argument for causality. We believe this is a significant contribution of the analysis. We also stratified by ZIP Code of origin, so the analysis is effectively a within ZIP Code of origin analysis looking at people from the same neighborhood who moved to places with different air pollution concentrations. We believe this is an important strength, which also controls for unmeasured confounders at the neighborhood level in the ZIP Code of origin.

To further our ability to draw causal inference, we also fit a propensity score model, controlling for both individual variables and contextual variables at the new ZIP Code. We believe the control for multiple preexisting diseases is rare in cohort studies of air pollution. This approach, if assumptions of positivity and exchangeability are met, provides causal estimates of the effects of change in PM_2.5_ exposure on mortality. Models restricted to exposures below the current EPA standard showed the same trend as models using all exposures (higher in whites and lower in blacks). Which is not surprising given that 60% of the sample were exposed to PM_2.5_ concentrations below 12 µg/m^3^. Hence this study provides strong evidence both for a causal association and for an association at low concentrations.

Another key aspect of this analysis is the focus on change in exposure. Comparing change in exposure to change in mortality is analogous to a difference in differences analysis. In addition, because stratification on original ZIP Code controls for past exposure before moving, the results of this analysis provide evidence that the effect of annual PM_2.5_ on annual mortality rates reported in other studies does not primarily reflect exposure over many years in the past, but truly indicates the effect of that year’s exposure. This is important because many risk analyses for interventions, such as EPA’s regulatory impact assessments, assume that the health benefits of reducing exposure will not be achieved within a year, but will be spread out over many years.

A recent meta-analysis^21^ of 53 studies estimated a nonlinear concentration-response with an overall HR of 1.13 at a concentration of 10 µg/m^3^, which is the median concentration in this cohort. However, they also estimated that the effect size in elderly cohorts such as this one was higher, which would yield an HR of 1.16. This is between our estimate for whites and blacks. Another publication^22^ among persons with relatively low PM_2.5_ exposures (mean exposure = 6.31 µg/m^3^) in Canada reported an HR of 1.26. Hence, these results are consistent with the prevailing literature of observational studies, while applying causal modeling techniques.

Our finding that whites are more vulnerable to change in air pollution exposure in this population is novel. Baseline mortality among blacks is higher than among whites, which can result in smaller relative effects but similar absolute effects when a proportionate hazard model is fit. Overall, whites were slightly older (mean age among whites was 77.4 compared to 76.0 among blacks) and increased susceptibility by age might explain some of the racial difference.

Key advantages of this study include that the entire national Medicare population who moved was studied, including people living in smaller cities, towns, and rural areas that have been underrepresented in most air pollution cohort studies; the large population included in the study; and the use of causal modeling techniques. Hence it provides evidence of likely causal effects, and at low concentrations below currently acceptable limits.

Support for a causal interpretation of these effect estimates comes from the vast toxicological literature on PM_2.5_ that has developed over the last 2 decades. In experimental studies PM_2.5_ has been shown to increase atherosclerosis,^23,24^ decrease the stability of atherosclerotic plaques,^25^ increase systemic inflammation and oxidative stress,^26^ worsen the response to ischemia,^27^ produce proarrhythmic changes in electrocardiograms, increase blood pressure,^28^ impair lung clearance of bacteria,^29^ and increase lung inflammation.^30^

Human intervention studies also support this conclusion. A randomized trial of air filtration versus sham filtration in dorm rooms of 55 college students for 9 days showed filtration reduced PM_2.5_ concentrations and reduced cortisol, cortisone, epinephrine, norepinephrine, glucose, insulin resistance, and blood pressure.^31^ A randomized trial of home air filtration reported improvements in microvascular function following a 48-hour exposure to filtered air versus sham filtration (unfiltered air).^32^ In another study, 200 participants were randomized to a particle filter versus a sham filter for a year.^33^ The sham filter resulted in a 7.8 mmHg higher systolic blood pressure in residents of those homes compared to the people with real particle filters. In yet another trial, 50 healthy subjects exposed to air from a busy street (PM_2.5_ = 24 μg/m^3^) versus filtered air (PM2.5 = 3 μg/m^3^) for 5 hours had a 25% reduction in nitroglycerin-induced vasodilation, increased sympathetic tone, and decreased parasympathetic tone at the higher concentration.^34^ Another study had subjects walk the streets of Beijing for 2 hours twice, once wearing a particle-filtering mask. Blood pressure was measured continuously and was lower when wearing the filter.^35^

One potential limitation of this analysis is that we do not have information on individual-level covariates such as smoking history or medical conditions with no hospitalization. However, in their analysis of risk of all-cause mortality in a subset of about 35,000 Medicare recipients for whom individual-level data were available, Makar et al^36^ showed that the estimates did not differ significantly when these individual-level covariates were included. In addition, Di et al^8^ showed that there was no or very little association between individual behavioral risk factors including smoking and body mass index, and PM_2.5_ exposure in a large subset of about 57,000 Medicare recipients indicating that omitting them would not significantly confound the association. Additionally, by stratifying on ZIP Code before move, we are comparing movers who likely have similar individual-level SEP thus controlling for some pre-move individual-level confounders. Finally, individual covariates are unlikely to be confounders in this analysis. High blood pressure in an individual does not produce air pollution. Confounding can only occur if neighborhoods with higher prevalence of high blood pressure also have higher air pollution. This can happen if an antecedent of high blood pressure (e.g., SES) predicts both neighborhood clustering and neighborhood air pollution. Hence it is primarily neighborhood-level covariates that are potential confounders. Again, stratifying on original neighborhood should control for all measured and unmeasured confounders there, and the IP weights likely control for the antecedents of such variables at the new neighborhood.

A second limitation of this study is exposure misclassification due to the use of modeled PM_2.5_ and potential incorrect ZIP Code assignment; if the ZIP Code reported in our dataset for billing purposes is not the ZIP Code of residence then the mover will be assigned the wrong exposure. We expect the first source of misclassification to be random with regards to exposure and outcome which, because our predicted exposure is monotonically increasing with measured exposure,^16^ biases our results to the null.^37,38^ The second source of misclassification is reduced by our exclusion of the year the move occurred, and is also unlikely to be differential. Finally, the use of the ZIP code centroid to assign exposure is not ideal as area-weighted methods or even residential exposures would have been preferred. Residential data are unfortunately not available for this population; however, area weighted exposures should be considered in future studies.

We note that when movers change residence, their PM_2.5_ exposure level is not the only factor that changes but so do other neighborhood characteristics of the new ZIP Code that influence mortality^39^ some of which may be correlated with air pollution. For example, it is possible that of 2 movers who initially live in the same ZIP Code, the one with lower SEP ends up in a neighborhood with higher air pollution compared to the one with higher SEP. In this scenario, SEP would confound the PM_2.5_-mortality relationship. However, if the person with lower SEP moved to a neighborhood with higher pollution because property values are lower in neighborhoods with higher air pollution, then by controlling for ZIP Code level covariates like median household value, and similarly for median income, etc. we control for this confounding.

The strengths of our analysis include the use of multiple causal modeling approaches which provide considerable support for a causal inference. The focus on change in pollution rather than pollution level alone reduces potential confounding, the focus on movers appears to randomize measured covariates, and likely unmeasured ones as well, the focus on within ZIP Code of origin analyses eliminates confounding by all measured or unmeasured covariates at the original ZIP Code, and the use of IP weights to control for confounding by covariates at the new ZIP Code, when combined suggest that unmeasured confounding is unlikely. Another strength is the large number of persons available in our analysis which allowed for precise estimates. Finally, the finding that the hazard ratio is still greater than 1 at exposures below 12 µg/m^3^ indicates that the PM_2.5_ standard needs to be lowered further to protect this older and more susceptible population.
